# 2,2,2-Tri­fluoro-*N*-(2-iodo­phen­yl)acetamide

**DOI:** 10.1107/S1600536813029851

**Published:** 2013-11-06

**Authors:** Yang Ruchun, Zhang Hui, Cao BanPeng

**Affiliations:** a Jiangxi Key Laboratory of Organic Chemistry, Jiangxi Science & Technology Normal University, Nanchang 330013, People’s Republic of China

## Abstract

The three F atoms in the title compound, C_8_H_5_F_3_INO, are disordered over two sets of sites [relative occupancies = 0.615 (14):0.385 (14)]. In the crystal, mol­ecules are linked by N—H⋯O hydrogen bonds, forming chains running along the *c*-axis direction. The dihedral angle between the ring and the amide group is 62.1 (3)°.

## Related literature

For effects of flourine on the properties of compounds, see: Jeschke (2004 ▶); Mueller *et al.* (2007 ▶); Purser *et al.* (2008 ▶). For the synthesis, see: Konfink *et al.* (2007 ▶).
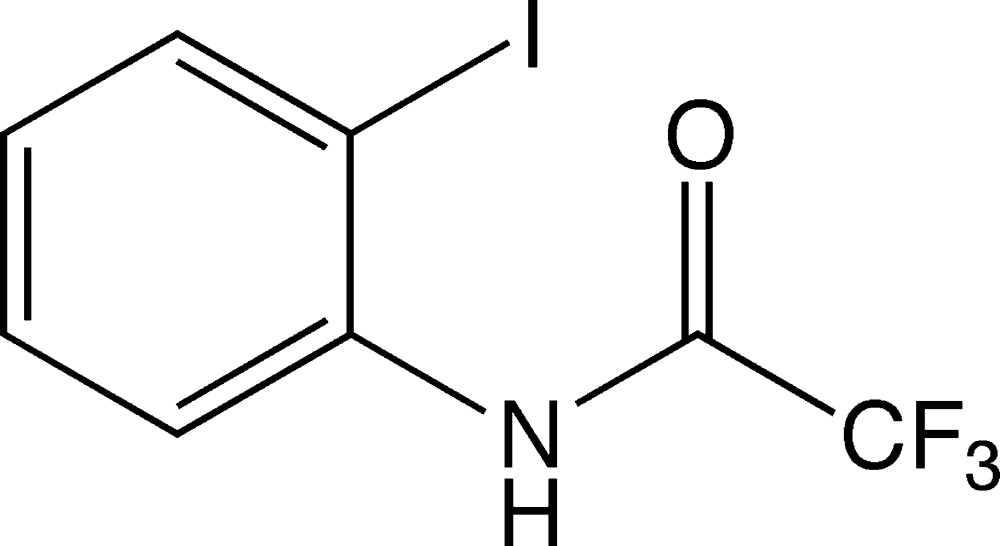


## Experimental

### 

#### Crystal data

C_8_H_5_F_3_INO
*M* *_r_* = 315.02
Tetragonal, 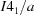

*a* = 15.8871 (1) Å
*c* = 15.9300 (2) Å
*V* = 4020.7 (6) Å^3^
*Z* = 16
Mo *K*α radiation
μ = 3.20 mm^−1^
*T* = 293 K
0.30 × 0.20 × 0.20 mm

#### Data collection

Agilent Xcalibur (Eos, Gemini) diffractometer
Absorption correction: multi-scan (*CrysAlis PRO*; Agilent, 2011 ▶) *T*_min_ = 0.447, *T*_max_ = 0.567
6063 measured reflections
1775 independent reflections
1153 reflections with *I* > 2σ(*I*)
*R*_int_ = 0.055

#### Refinement

*R*[*F* ^2^ > 2σ(*F* ^2^)] = 0.044
*wR*(*F* ^2^) = 0.105
*S* = 1.06
1775 reflections
155 parameters
450 restraints
H-atom parameters constrained
Δρ_max_ = 0.42 e Å^−3^
Δρ_min_ = −0.54 e Å^−3^

### 

Data collection: *CrysAlis PRO* (Agilent, 2011 ▶); cell refinement: *CrysAlis PRO*; data reduction: *CrysAlis PRO*; program(s) used to solve structure: *SHELXS97* (Sheldrick, 2008 ▶); program(s) used to refine structure: *SHELXL97* (Sheldrick, 2008 ▶); molecular graphics: *SHELXTL* (Sheldrick, 2008 ▶); software used to prepare material for publication: *SHELXTL*.

## Supplementary Material

Crystal structure: contains datablock(s) I, global. DOI: 10.1107/S1600536813029851/ff2122sup1.cif

Structure factors: contains datablock(s) I. DOI: 10.1107/S1600536813029851/ff2122Isup2.hkl

Supplementary material file. DOI: 10.1107/S1600536813029851/ff2122Isup3.cml

Additional supplementary materials:  crystallographic information; 3D view; checkCIF report

## Figures and Tables

**Table 1:** Hydrogen-bond geometry (Å, °)

*D*—H⋯*A*	*D*—H	H⋯*A*	*D*⋯*A*	*D*—H⋯*A*
N1—H1⋯O1^i^	0.86	2.08	2.917 (6)	163

Symmetry code: (i) 
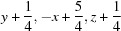
.

**Fig. 1.**
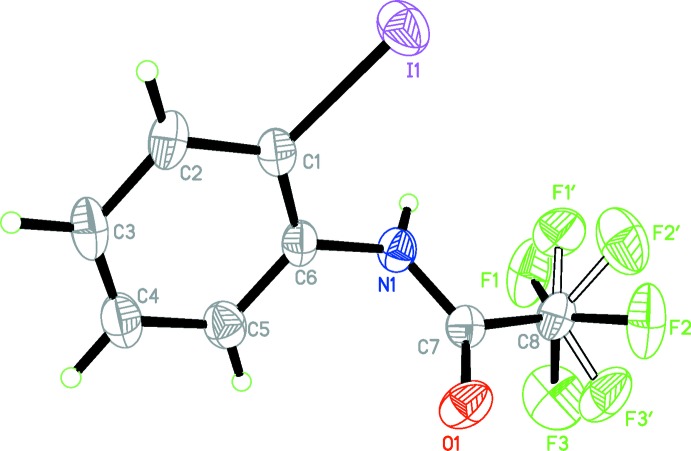
Thermal ellipsoid plot of C8H5F3INO. Ellipsoids are drawn at the 30% probability level and H atoms are represented as small spheres of arbitrary radius.

## supplementary crystallographic information

### 1. Comment

Aromatic compounds play an important role in the pharmaceutical and agrochemical industries as important intermediates. The fluorine atom has a strong electron withdrawing character and small atomic radius. Introduction of one or more F atoms can change the physiological activity, pharmacological activity and metabolic stability properties of the compound (Jeschke, 2004; Mueller, *et al.*, 2007; Purser, *et al.*, 2008). In our study, we report an aromatic compounds containing fluorine, 2,2,2-trifluoro-N-(2-iodophenyl)acetamide, which could be used in the synthesis of many bioactive compounds.

There is a single molecule in the asymmetric unit of the title compound 2,2,2-trifluoro-N-(2-iodophenyl)acetamide, C_8_H_5_F_3_INO. In the crystal, The three F atoms are disordered over two sites [relative occupancies 0.615 (14):0.385 (14)]. The crystal packing is stabilized by N1—H1···O1 hydrogen bond that connect molecules into chains running along the *c* direction.

### 2. Experimental

The title compound was synthesized according to the previously reported procedure (Konfink, *et al.*, 2007).

### 3. Refinement

H atoms bond to N were located in a difference map and refined with N—H = 0.86 Å and *U*_iso_(H) = 1.2*U*_eq_(N) other H atoms attached to C were fixed geometrically and treated as riding with C—H = 0.96 Å (methyl) or 0.93 Å (aromatic) and with *U*_iso_(H) = 1.2*U*_eq_(aromatic) or *U*_iso_(H) = 1.5*U*_eq_(methyl).

### Crystal data

**Table:**

C_8_H_5_F_3_INO	*D*_x_ = 2.075 Mg m^−^^3^
*M**_r_* = 315.02	Mo *K*α radiation, λ = 0.71073 Å
Tetragonal, *I*4_1_/*a*	Cell parameters from 1114 reflections
Hall symbol: -I 4ad	θ = 3.1–21.8°
*a* = 15.8871 (1) Å	µ = 3.20 mm^−^^1^
*c* = 15.9300 (2) Å	*T* = 293 K
*V* = 4020.7 (6) Å^3^	Block, colourless
*Z* = 16	0.30 × 0.20 × 0.20 mm
*F*(000) = 2368	

### Data collection

**Table:**

Agilent Xcalibur (Eos, Gemini) diffractometer	1775 independent reflections
Radiation source: fine-focus sealed tube	1153 reflections with *I* > 2σ(*I*)
Graphite monochromator	*R*_int_ = 0.055
ω scans	θ_max_ = 25.0°, θ_min_ = 3.1°
Absorption correction: multi-scan (*CrysAlis PRO*; Agilent, 2011)	*h* = −17→15
*T*_min_ = 0.447, *T*_max_ = 0.567	*k* = −18→12
6063 measured reflections	*l* = −18→18

### Refinement

**Table:**

Refinement on *F*^2^	Primary atom site location: structure-invariant direct methods
Least-squares matrix: full	Secondary atom site location: difference Fourier map
*R*[*F*^2^ > 2σ(*F*^2^)] = 0.044	Hydrogen site location: inferred from neighbouring sites
*wR*(*F*^2^) = 0.105	H-atom parameters constrained
*S* = 1.06	*w* = 1/[σ^2^(*F*_o_^2^) + (0.0324*P*)^2^ + 3.0148*P*] where *P* = (*F*_o_^2^ + 2*F*_c_^2^)/3
1775 reflections	(Δ/σ)_max_ < 0.001
155 parameters	Δρ_max_ = 0.42 e Å^−^^3^
450 restraints	Δρ_min_ = −0.54 e Å^−^^3^

### Special details

**Table:**

Geometry. All esds (except the esd in the dihedral angle between two l.s. planes) are estimated using the full covariance matrix. The cell esds are taken into account individually in the estimation of esds in distances, angles and torsion angles; correlations between esds in cell parameters are only used when they are defined by crystal symmetry. An approximate (isotropic) treatment of cell esds is used for estimating esds involving l.s. planes.
Refinement. Refinement of F^2^ against ALL reflections. The weighted R-factor wR and goodness of fit S are based on F^2^, conventional R-factors R are based on F, with F set to zero for negative F^2^. The threshold expression of F^2^ > 2sigma(F^2^) is used only for calculating R-factors(gt) etc. and is not relevant to the choice of reflections for refinement. R-factors based on F^2^ are statistically about twice as large as those based on F, and R- factors based on ALL data will be even larger.

### Fractional atomic coordinates and isotropic or equivalent isotropic displacement parameters (Å^2^)

**Table:**

	*x*	*y*	*z*	*U*_iso_*/*U*_eq_	Occ. (<1)
I1	0.61704 (3)	0.35482 (3)	0.43754 (3)	0.0704 (2)	
N1	0.7883 (3)	0.4465 (3)	0.5004 (3)	0.0465 (12)	
H1	0.7771	0.4175	0.5446	0.056*	
O1	0.8853 (3)	0.4661 (3)	0.3977 (2)	0.0627 (13)	
F1	0.9082 (8)	0.3600 (9)	0.5849 (6)	0.095 (4)	0.615 (14)
F2	0.9342 (9)	0.3088 (7)	0.4454 (6)	0.098 (4)	0.615 (14)
F3	0.9903 (8)	0.4012 (8)	0.5219 (9)	0.109 (4)	0.615 (14)
F1'	0.8791 (11)	0.3219 (12)	0.5583 (13)	0.081 (5)	0.385 (14)
F2'	0.8837 (13)	0.2878 (9)	0.4878 (15)	0.099 (5)	0.385 (14)
F3'	0.9922 (12)	0.3650 (14)	0.4827 (12)	0.091 (5)	0.385 (14)
C1	0.6481 (4)	0.4831 (4)	0.4479 (3)	0.0468 (15)	
C2	0.5885 (4)	0.5432 (4)	0.4246 (4)	0.0594 (18)	
H2	0.5342	0.5272	0.4099	0.071*	
C3	0.6121 (5)	0.6273 (4)	0.4237 (4)	0.0649 (19)	
H3	0.5728	0.6682	0.4094	0.078*	
C4	0.6921 (5)	0.6507 (5)	0.4434 (4)	0.0634 (18)	
H4	0.7076	0.7071	0.4400	0.076*	
C5	0.7503 (4)	0.5913 (4)	0.4685 (4)	0.0543 (16)	
H5	0.8045	0.6079	0.4830	0.065*	
C6	0.7280 (4)	0.5074 (4)	0.4720 (3)	0.0440 (14)	
C7	0.8600 (4)	0.4334 (4)	0.4613 (3)	0.0431 (14)	
C8	0.9161 (5)	0.3650 (5)	0.5022 (5)	0.0577 (18)	

### Atomic displacement parameters (Å^2^)

**Table:**

	*U*^11^	*U*^22^	*U*^33^	*U*^12^	*U*^13^	*U*^23^
I1	0.0683 (4)	0.0521 (4)	0.0909 (4)	−0.0005 (2)	−0.0240 (3)	0.0036 (3)
N1	0.052 (3)	0.047 (3)	0.041 (2)	0.010 (2)	−0.001 (2)	0.008 (2)
O1	0.055 (3)	0.086 (4)	0.047 (2)	0.011 (3)	0.007 (2)	0.018 (2)
F1	0.112 (8)	0.113 (8)	0.061 (5)	0.062 (6)	−0.008 (5)	0.010 (5)
F2	0.129 (8)	0.082 (6)	0.083 (5)	0.057 (6)	−0.001 (6)	−0.014 (5)
F3	0.077 (6)	0.118 (8)	0.133 (8)	0.006 (6)	−0.065 (6)	0.013 (6)
F1'	0.084 (9)	0.087 (10)	0.073 (9)	0.015 (7)	0.017 (7)	0.041 (8)
F2'	0.112 (10)	0.066 (8)	0.118 (10)	0.016 (7)	−0.039 (9)	0.003 (8)
F3'	0.063 (8)	0.116 (11)	0.092 (9)	0.036 (8)	0.018 (8)	0.027 (8)
C1	0.055 (4)	0.040 (3)	0.045 (3)	0.008 (3)	0.002 (3)	−0.001 (3)
C2	0.055 (4)	0.058 (4)	0.065 (4)	0.022 (3)	−0.007 (3)	−0.001 (3)
C3	0.080 (5)	0.046 (4)	0.069 (4)	0.026 (4)	−0.007 (4)	0.005 (3)
C4	0.078 (5)	0.045 (4)	0.067 (4)	0.013 (4)	0.004 (4)	−0.001 (3)
C5	0.066 (4)	0.048 (4)	0.049 (3)	0.003 (3)	0.004 (3)	−0.001 (3)
C6	0.053 (4)	0.042 (3)	0.037 (3)	0.014 (3)	0.006 (3)	0.004 (3)
C7	0.047 (4)	0.045 (4)	0.037 (3)	0.000 (3)	−0.005 (3)	−0.005 (3)
C8	0.052 (4)	0.062 (5)	0.059 (4)	0.015 (4)	−0.002 (4)	−0.004 (4)

### Geometric parameters (Å, º)

**Table:**

I1—C1	2.103 (6)	C1—C6	1.382 (8)
N1—C7	1.316 (7)	C1—C2	1.394 (8)
N1—C6	1.433 (7)	C2—C3	1.389 (9)
N1—H1	0.8600	C2—H2	0.9300
O1—C7	1.208 (6)	C3—C4	1.361 (9)
F1—C8	1.326 (12)	C3—H3	0.9300
F2—C8	1.303 (11)	C4—C5	1.380 (9)
F3—C8	1.349 (13)	C4—H4	0.9300
F1'—C8	1.271 (16)	C5—C6	1.381 (8)
F2'—C8	1.351 (16)	C5—H5	0.9300
F3'—C8	1.248 (18)	C7—C8	1.548 (9)
			
C7—N1—C6	122.5 (5)	C5—C6—C1	119.6 (6)
C7—N1—H1	118.8	C5—C6—N1	119.6 (6)
C6—N1—H1	118.8	C1—C6—N1	120.8 (6)
C6—C1—C2	120.4 (6)	O1—C7—N1	128.1 (6)
C6—C1—I1	120.5 (4)	O1—C7—C8	117.6 (6)
C2—C1—I1	118.9 (5)	N1—C7—C8	114.3 (6)
C3—C2—C1	118.6 (7)	F3'—C8—F1'	128.5 (12)
C3—C2—H2	120.7	F2—C8—F1	132.0 (10)
C1—C2—H2	120.7	F2—C8—F3	105.1 (10)
C4—C3—C2	120.8 (7)	F1—C8—F3	82.9 (11)
C4—C3—H3	119.6	F3'—C8—F2'	109.1 (13)
C2—C3—H3	119.6	F1'—C8—F2'	56.9 (16)
C3—C4—C5	120.4 (7)	F3'—C8—C7	116.9 (10)
C3—C4—H4	119.8	F1'—C8—C7	114.1 (9)
C5—C4—H4	119.8	F2—C8—C7	108.4 (6)
C4—C5—C6	120.0 (7)	F1—C8—C7	113.9 (7)
C4—C5—H5	120.0	F3—C8—C7	107.6 (8)
C6—C5—H5	120.0	F2'—C8—C7	110.3 (8)

### Hydrogen-bond geometry (Å, º)

**Table:**

*D*—H···*A*	*D*—H	H···*A*	*D*···*A*	*D*—H···*A*
N1—H1···O1^i^	0.86	2.08	2.917 (6)	163

Symmetry code: (i) *y*+1/4, −*x*+5/4, *z*+1/4.
